# Antifungal activity of indolicidin-derived peptide In-58 against *Sporothrix globosa in vitro* and *in vivo*

**DOI:** 10.3389/fmed.2024.1458951

**Published:** 2024-09-12

**Authors:** Sushan Li, Zhanhan Tang, Zhe Liu, Sha Lv, Chunli Yao, Shuang Wang, Fuqiu Li

**Affiliations:** Department of Dermatology, The Second Hospital of Jilin University, Changchun, China

**Keywords:** *Sporothrix globosa*, antimicrobial peptide, indolicidin derivative, antifungal activity, *Galleria mellonella*

## Abstract

In-58, a peptide derived from indolicidin, shows extraordinary antibacterial activity and lower toxicity than indolicidin toward mammalian cells. Here, we investigated the antifungal activity of In-58 against the human pathogen *Sporothrix globosa in vitro* and *in vivo*. In-58 markedly inhibited the growth of *Sporothrix globosa* isolates in microdilution assays and showed no antagonism with any tested antifungal agent (itraconazole, terbinafine or amphotericin B). Scanning electron microscopy and propidium iodide staining indicated that In-58 alters the cell wall integrity and interacts with DNA, leading to disruption of *S. globosa* in a dose-dependent manner. In *S. globosa*, the mitochondrial membrane potential decreased and reactive oxygen species increased after treatment with In-58. *In vivo* experiments in the *Galleria mellonella* (greater wax moth) larval infection model revealed the effectiveness of In-58 against *S. globosa* infection with low toxicity. Our results indicate that In-58 possesses remarkable antifungal activity against *S. globosa in vitro* and *in vivo*. It has potential as a novel drug for the treatment of sporotrichosis.

## 1 Introduction

Sporotrichosis is one of the most common subcutaneous mycoses. It is acquired by traumatic inoculation of contaminated soil or plants, or by zoonotic transmission via bites and scratches from infected animals; it is emerging as a threat to humans and animals worldwide (1, 2). *Sporothrix* is a genus containing 53 species, among which *Sporothrix brasiliensis*, *Sporothrix schenckii*, *Sporothrix globosa*, and *Sporothrix luriei* cause cutaneous infection in humans and other mammalian hosts (3). *S. globosa* is the most common causative fungus of human sporotrichosis in China, especially northeast China (4); it is also distributed in other countries worldwide, including in Europe (the United Kingdom, Spain, Italy), North America (the United States, Mexico, and Guatemala), South America (Columbia), and Asia (India, Japan) (5, 6). The treatment options for sporotrichosis are limited, with only a few drugs available, mainly comprising potassium iodide, itraconazole (ITZ), terbinafine (TRB) and amphotericin B (AMB). However, these drugs have harmful side-effects, such as hyperthyroidism or hypothyroidism for potassium iodide, and epigastric pain and nausea for ITZ (7). The limited medication options, their side-effects, and the emergence of drug-resistant strains require the development of new methods to treat sporotrichosis (8). Emerging approaches include physical therapies such as photodynamic therapy (9) and heat therapy (10), and novel medications such as natural extracts (11), chemical compounds (12), and antifungal peptides (AFPs) (13).

Antimicrobial peptides (AMPs) were originally identified as exerting activity via membrane permeabilization, AMPs with antifungal activity may exhibit enhanced affinity for the phospholipids in fungal membranes, specifically phosphatidylserine and phosphatidylethanolamine which suggests a unique relationship between their structure and activity. The barrel-stave, carpet-like and toroidal models used to describe the damage AMPs dose to bacterial membrane are also well-evidenced for fungal walls. However, the specific antifungal mechanism of AMPs is generally more complex, indicating the necessity for deeper research (14, 15). Compared with traditional antimicrobial drugs, AMPs have broad-spectrum activity and are not subject to microbial drug resistance. AFPs play crucial roles in combating fungal infection. AFPs can be found from a diverse range of sources, including plants, mammals, amphibians, insects, crabs, spiders, and fungi (16, 17). AFPs can be classified into different types, including proline-rich, tryptophan- and arginine-rich, histidine-rich, and glycine-rich peptides. Indolicidin belongs to the tryptophan- and arginine-rich type. It was initially purified from the cytoplasmic granules of bovine neutrophils in 1992 (18). Indolicidin is a promising AMP with an incredibly wide spectrum of biological activity, including against bacteria, fungi, viruses, and parasites (19). In-58 is an AMP derived from indolicidin, created by replacing all the tryptophan residues with D-phenylalanine, which improves its antibacterial activity while decreasing its toxicity toward mammalian cells (20). However, the antifungal ability of In-58 remains to be tested.

In this study, we examined the susceptibility of *S. globosa* to In-58 alone and in combination with other antifungal agents. In-58 showed excellent inhibition of fungal growth without antagonistic effects with the other tested drugs. The mechanism of In-58 against *S. globosa* was studied by observing the ultrastructure of *S. globosa* treated with different concentrations of In-58 using scanning electron microscopy (SEM). Elevated reactive oxygen species (ROS) accumulation and a decreased mitochondrial membrane potential (MMP) were observed after In-58 treatment. *In vivo* experiments using the *Galleria mellonella* (greater wax moth) larval infection model showed the effectiveness of In-58 against *S. globosa* infection with comparatively low toxicity. Overall, our results reveal the antifungal activities of a novel indolicidin derivative, In-58, against *S. globosa* and potentially provide a new perspective for treatment of sporotrichosis.

## 2 Materials and methods

### 2.1 Fungal strains and culture

Fungal strains were isolated from patients diagnosed with sporotrichosis in The Department of Dermatology, The Second Hospital of Jilin University, Changchun, Jilin, China and identified as *S. globosa* by sequencing the calmodulin gene and comparison of gene sequences with published data. GenBank accession codes for strains are shown in Table 1. The isolates were inoculated on potato–dextrose–agar (PDA) at 25°C for 1–2 weeks before use. *Candida parapsilosis* ATCC 22019 and *Candida krusei* ATCC 6258 were used as reference isolates for MIC determination and drug interaction assays, provided by the Department of Pathogenobiology, Jilin University Mycology Research Center, Changchun, Jilin, China. The isolates used in this study are listed in Table 1.

**Table 1:** Twenty one isolates and reference isolates used in the study.

Isolate ID	GenBank accession code	BLAST score for isolate identification	Age	Host gender	Clinical type
SHJU10001	OR900858	1,447	65	M	L
SHJU10003	OR900859	1,428	46	M	F
SHJU10004	OR900860	1,428	42	F	F
SHJU10005	OR900861	1,428	73	M	F
SHJU10006	OR900862	1,434	58	F	F
SHJU10007	OR900863	1,434	61	M	F
SHJU10008	OR900864	1,434	50	M	F
SHJU10009	OR900865	1,434	55	F	L
SHJU10010	OR900866	1,439	42	M	L
SHJU10020	OR900874	1,437	75	M	F
SHJU10021	OR900875	1,437	55	F	F
SHJU10022	OR900876	1,487	54	F	F
SHJU10023	OR900877	1,413	60	F	F
SHJU10024	OR900878	1,415	37	F	F
SHJU10025	OR900879	1,474	64	F	F
SHJU10026	OR900880	1,424	50	F	F
SHJU10027	OR900881	1,428	43	F	F
SHJU10028	OR900845	1,356	63	F	L
SHJU10029	OR900846	1,430	50	F	F
SHJU10030	OR900847	1,432	57	M	F
SHJU10031	OR900848	1,434	72	F	L
Reference isolate	*Candida krusei* ATCC6258				
	*Candida parapsilosis* ATCC 22019				

### 2.2 Antifungal peptide

Peptide In-58 [NH_2_–(CH_2_)_10_–CO–ILP(D)FK(D)FP(D)F(D)FP(D)FRR–NH_2_], a novel synthetic analogue of indolicidin (NH_2_–ILPWKWPWWPWRR–NH_2_), was synthesized commercially (Taijia Biotech Co., Ltd., Hangzhou, China), resuspended in sterile distilled water (final stock concentration 1 mg/mL), and stored at −20°C until use. In the peptide sequence above, “(D)F” indicates D-phenylalanine.

### 2.3 Determination of minimum inhibitory concentrations

Antifungal susceptibility testing was performed for 21 *S. globosa* isolates to determine minimum inhibitory concentrations (MICs) by the broth microdilution method as outlined in The Clinical & Laboratory Standards Institute (CLSI) reference standard M38-ed3 (21) for filamentous fungi. To evaluate the *in vitro* susceptibility of *S. globosa* to In-58, ITZ (Macklin, Shanghai, China), TRB (Macklin), and AMB (Macklin), assays were performed in 96-well round-bottomed microplates. ITZ, TRB, and AMB were first diluted in dimethyl sulfoxide (Solarbio, Beijing, China) while In-58 was resuspended in sterile distilled water. Drugs were finally diluted in RPMI-1640 medium (Gibco, Thermo Fisher Scientific, Waltham, Massachusetts, United States) buffered to pH 7.2 with 0.165 M morpholinepropanesulfonic acid. The drugs were tested at 10 concentrations (0.06–32 μg/mL). The numbers of suspended conidia were adjusted to final concentrations of 1 × 10^4^ to 5 × 10^4^ colony-forming units (CFU)/mL in RPMI-1640 medium. The wells of the microplates were filled with 100 μL of inoculum and 100 μL of drug equivalent to the final concentration indicated in the test and the plates were incubated at 35°C for 72 h. The viabilities of the inocula were tested by plating dilutions of the suspensions on PDA plates. The MICs for ITZ, TRB and AMB were defined as the lowest concentrations that resulted in complete inhibition of growth, and for In-58 as the lowest concentration that resulted in 50% inhibition of growth. To determine minimum fungicidal concentrations (MFCs), suspensions from wells containing drug at above the MIC were plated on PDA. The MFC was considered the lowest drug concentration that killed >99.9% of fungal cells. A drug is considered to have a fungicidal effect if the MFC is ≤4× the MIC (22). The tests were repeated three times.

### 2.4 Drug interaction assays

Drug interaction between In-58 and ITZ, TRB or AMB was evaluated by checkerboard assay for 10 *S. globosa* isolates in 96-well microplates, according to a previous protocol (23). Briefly, 50 μL of In-58 solution (final tested concentration 0.13–16 μg/mL) was distributed in the columns, and 50 μL of antifungal drug solution (ITZ, 0.13–16 μg/mL; TRB, 0.03–4 μg/mL; or AMB, 0.13–16 μg/mL) in the rows, to give a checkerboard matrix. Then, 100 μL of standardized inoculum was added to the wells. Microplates were incubated for 72 h at 35°C for visual reading of MICs as described in Section 2.3. The assay was performed at least three times.

The fractional inhibitory concentration index (FICI) was calculated according to the formula:

$$\begin{array}{l} \mathrm{FICI}={FIC}_{\mathrm{Drug}}+{FIC}_{\mathrm{Peptide}}=\\ \;\frac{{\mathrm{MIC}}_{\mathrm{Drug}}\;\mathrm{in}\;\mathrm{combination}}{{\mathrm{MIC}}_{\mathrm{Drug}}\;\mathrm{alone}}+\frac{{\mathrm{MIC}}_{\mathrm{Peptide}}\;\mathrm{in}\;\mathrm{combination}}{\;{\mathrm{MIC}}_{\mathrm{Peptide}}\mathrm{alone}} \end{array}$$

FICI values were determined for In-58 in combination with ITZ, TRB and AMB, and interpreted as follows: synergy if FICI ≤0.5, antagonism if FICI >4.0, indifferent if FICI is within the range of 0.5–4.0 (24).

### 2.5 Time-kill kinetics

The experiment was performed in accordance with a previous report (25) in shaken-flasks and using Sabouraud dextrose broth. In-58 (1, 2, 4, and 8 μg/mL) was evaluated against *S. globosa* SHJU10001. Adjusted inoculum (final concentration 4 × 10^5^ cells/mL) in the exponential phase was added and the flasks were incubated aerobically at 25°C with shaking at 150 rpm for 48 h. The control used was phosphate-buffered saline (PBS) with the same amount of inoculum. The absorbance at 530 nm was measured after extracting 1 mL from each flask at 0, 2, 4, 6, 8, 12, 24, 36, and 48 h. The experiment was performed in triplicate.

### 2.6 Micromorphological observation of *Sporothrix globosa* following treatment with In-58

Slide culture was obtained by inoculating *S. globosa* SHJU10001 on PDA with different concentrations of In-58 (4, 8 μg/mL) and incubating for 5–7 days at 25°C. PDA without In-58 was used as the negative control. The configuration of mycelia and conidia was observed by microscopy (Nikon Alphaphot-2 YS2, Tokyo, Japan) to determine the effects of In-58 on the growth of *S. globosa*.

SEM was used to study the effect of In-58 on the cell morphology of *S. globosa* SHJU10001. Preparation of samples was carried out based on the publication by Bello et al. (26). Slide culture of *S. globosa* was conducted for 7 days and the fungus was then incubated with In-58 (4 or 8 μg/mL) at 25°C for 12 h in a six-well plate. Post-incubation, aliquots were withdrawn, washed with PBS, and fixed with 2.5% glutaraldehyde for 4 h at room temperature. Afterward, fixed cells were washed with PBS and subjected to gradient dehydration with ethanol (30%, 10 min, 50%, 10 min, 70%, 10 min, 90%, 10 min; 100%, 30 min) and air-dried at room temperature. Dehydrated cells were used to prepare slides, gold-coated using a MC1000 Ion Sputter Coater (Hitachi, Japan), and observed under a scanning electron microscope (Zeiss Gemini 2, Germany).

### 2.7 Propidium iodide staining

*S. globosa* SHJU10001 cells (approximately 10^5^–10^6^ CFU/mL) in the exponential phase were treated with 4 or 8 μg/mL of In-58 for 12 h at 25°C. PBS was used in negative controls. The *S. globosa* cells were harvested by centrifugation and washed using PBS. PI staining was performed following the staining protocol according to the manufacturer instructions (ST512, Beyotime Institute of Biotechnology, Jiangsu, China). Then, the cells were observed by fluorescence microscopy (Zeiss Axio Imager Z2, Germany). Fluorescence-positive cells from each group were quantified in at least three fields of view by using ImageJ software, and the count was averaged. The experiment was repeated at least three times.

### 2.8 Intracellular reactive oxygen species accumulation assay

The experiment was carried out according to the study of Li et al. (27). The production and accumulation of ROS in *S. globosa* cells were analyzed using dichlorofluorescein diacetate (DCFH-DA) as the fluorescent probe in a ROS detection assay kit (S0033S, Beyotime Institute of Biotechnology). After treatment with 4 or 8 μg/mL of In-58 as described in section 2.7, *S. globosa* SHJU10001 cells (approximately 10^6^–10^7^ CFU/mL) were harvested by centrifugation, washed, and resuspended in PBS. DCFH-DA was added to a final concentration of 10 μM. Following a 20 min incubation in the dark, the fluorescence intensity of *S. globosa* cells was determined using a NovoCyte Flow Cytometer (Agilent, United States). Software FlowJo was used for flow cytometry data analysis. The concentrated cell population was selected under pseudocolor conditions, removing cell debris located in the bottom left corner of the axes. Within the selected cell population, switch the display mode to histogram and change the horizontal axis to FITC. Comparing the peak values of the negative control and the experimental groups to determine the ROS-positive gate range, which serves as the gating strategy. The experiment was repeated at least three times.

### 2.9 Mitochondrial membrane potential

MMP was assessed using a JC-1 kit (Beyotime Institute of Biotechnology). According to Yan et al. (28), *S. globosa* SHJU10001 cells (approximately 10^6^–10^7^ CFU/mL) were treated with 4 or 8 μg/mL of In-58 for 12 h at 25°C. PBS was used in negative controls. The cells were harvested, washed, and stained with JC-1 (1×) for 20 min in the dark. After rinsing and resuspension in JC-1 buffer, the cells were analyzed by flow cytometry. Mitochondria containing green-fluorescent JC1 monomers in apoptotic cells were detectable in the FL1 channel, while red-fluorescent JC-1 aggregates in healthy cells were detected in the FL2 channel. The ratio of the fluorescence intensities of aggregates of JC-1 (FL2) to monomers (FL1) was calculated as previously described (29). The experiment was repeated at least three times.

### 2.10 *Galleria mellonella* survival curve assays

Final (6th) instar larvae of *G. mellonella* were purchased from Qingcheng Biotechnology, Beijing, China. The larvae (2–3 cm long, body weight 200–350 mg) were stored 15°C for up to 1 week before use in experiments to investigate the potential application of In-58 in treatment for sporotrichosis. Five groups of *G. mellonella* larvae were established to study the toxicity and antifungal efficacy of In-58. Larvae were swabbed at the injection site with 70% ethanol and injection of 10 μL was made 2–3 mm deep into the last right proleg with a 100 μL microsyringe (Gaoge, Shanghai, China) (30). The larvae were grouped as follows: Group I (PBS control); Group II (infection control, no drug treatment); Group III (infection +5 mg/kg In-58); Group IV (infection +20 mg/kg ITZ); Group V (no infection +5 mg/kg In-58). The PBS control group was used to ensure that death was not due to trauma. Group V was a toxicity test of In-58. In accordance with a previous study (31), 10^6^ CFU was determined as the inoculum dose; the *G. mellonella* were inoculated with *S. globosa* SHJU10001 suspended in PBS. In Groups III and IV, In-58 and ITZ were given, respectively, 1 h post-infection in another proleg of the larvae. Each group contained 30 larvae in three 9 cm Petri dishes, kept at 35°C. Survival was monitored daily for 10 days. Lack of reaction to physical stimuli and extensive melanization of the body were taken as indicators of death (32). Dead larvae were removed from the Petri dish and recorded.

### 2.11 Determination of fungal burden from infected *Galleria mellonella*

To monitor the fungal burden during different phases of infection, an experiment with another 10 inoculated larvae per group was conducted in the same conditions as for Groups I to V (see Section 2.10). The protocol was as per Reis et al. (32). Five larvae were randomly selected from the plate daily for 7 consecutive days, cleaned with 70% ethanol, and then a proleg of each larva was pierced with the needle of microsyringe and the hemolymph that emerged was collected using a pipette; 20 μL of hemolymph was collected from each individual, making a total of 100 μL from the five larvae. The hemolymph was immediately placed into a microcentrifuge tube containing insect physiological saline [IPS; 150 mM sodium chloride, 5 mM potassium chloride, 10 mM Tris–HCl (pH 6.9), 10 mM ethylenediaminetetraacetic acid, 30 mM sodium citrate] in a 1:10 proportion (33). Tenfold serial dilutions were plated on PDA containing 25 μg/mL chloramphenicol (Coolaber, Beijing, China); the plates were incubated for 7 days at 25°C. Colonies were counted for determination of fungal burden in the larvae and were confirmed as *Sporothrix* by lactophenol cotton blue (LPCB) staining.

### 2.12 Enumeration of hemocytes from *Galleria mellonella*

Larvae were inoculated in the conditions used for Groups I–V in section 2.10 (10 larvae/group); hemolymph was extracted as described in Section 2.11. Hemolymph (100 μL) was collected from five larvae from each group daily for 7 consecutive days. The hemolymph from each group of larvae was pooled and centrifuged at 700 × *g* for 5 min at 4°C. Pellets were washed twice with ice-cold IPS and collected by centrifugation at 700 × *g* for 5 min, then resuspended in ice-cold IPS in a 1:10 proportion (33). A total of 9 μL was withdrawn from suspension and mixed with 1 μL of trypan blue to enable cell enumeration in a hemocytometer. The experiment was performed in triplicate.

### 2.13 Histopathological examination of *Galleria mellonella*

To evaluate the effects of fungal infection and drug function (In-58 and ITZ) within host tissues, 10 infected larvae from each group (see Section 2.10) were incubated for up to 10 days at 35°C. Histopathological examination followed the protocol in the publication of Huang et al. (34). At the indicated time points, larvae were fixed by soaking in 4% buffered formalin. After fixation for 48 h, the whole larva was dissected sagittally into halves. Then, samples were dehydrated and embedded in paraffin, sectioned, and stained with hematoxylin and eosin or Calcofluor-White (CFW) stain.

### 2.14 Statistical analysis

Statistical analyses were performed, and survival curves were plotted using GraphPad Prism 10.2 software (GraphPad Software, San Diego, CA, United States). Statistical analyses of MIC50 and MFC50 were performed using Mann–Whitney tests. Statistical analyses of absorbance (λ = 530 nm), PI fluorescent positive cells, ROS accumulation, MMP, fungal burden were performed using One-way analysis of variance. Survival curves were plotted using Kaplan–Meier method, and statistical analyses were performed using the log-rank test for comparisons among groups. A *p*-value less than 0.05 was considered statistically significant.

## 3 Results

### 3.1 Determination of MICs of In-58

The MIC values determined for reference strains (*Candida* spp.) fell within the quality control range for antimicrobial susceptibility testing according to CLSI guidelines. Twenty-one isolates of *S. globosa* were also tested. In-58 showed antifungal activity against the mycelial phase of *S. globosa* with MICs ranging from 0.5 to 4 μg/mL and MFCs ranging from 1 to 8 μg/mL. The MICs of ITZ, TRB, and AMB were 1–4 μg/mL, 0.13–0.5 μg/mL, and 2–8 μg/mL, respectively, and the MFCs were 2–16 μg/mL, 0.25–1 μg/mL, and 4–16 μg/mL, respectively. The geometric mean (GM) MIC of 2.69 μg/mL for In-58, 1.87 μg/mL for ITZ, 0.20 μg/mL for TRB and 3.87 μg/mL for AMB. MFC GM of 5.21 μg/mL for In-58, 4.13 μg/mL for ITZ, 0.47 μg/mL for TRB and 8 μg/mL for AMB. Mann–Whitney tests were performed to determine median MICs and median MFCs. The MIC50 was 4 μg/mL for In-58, compared with 2 μg/mL for ITZ (*p* = 0.0271), 0.25 μg/mL for TRB (*p* < 0.0001), and 4 μg/mL for AMB (*p* = 0.0252). The MFC50 was 4 μg/mL for In-58, compared with 4 μg/mL for ITZ (*p* = 0.104), 0.5 μg/mL for TRB (*p* < 0.0001), and 8 μg/mL for AMB (*p* = 0.0053). Thus, in terms of MFC50 value, In-58 showed no significant difference when compared with ITZ, which means it may kill the fungus at the same concentration as ITZ, while In-58 had a lower MFC50 than AMB, which indicates better activity (Tables 2, 3).

**Table 2:** MIC and MFC profiles of In-58 and antifungals against mycelial phase of *S. globosa*.

*S. globosa* of SHJU ID no.	In-58		ITZ		TRB		AMB	
	MIC	MFC	MIC	MFC	MIC	MFC	MIC	MFC
10,001	4	8	2	4	0.25	0.5	8	16
10,003	4	8	1	2	0.25	1	4	8
10,004	4	8	2	4	0.25	0.5	4	8
10,005	4	8	1	4	0.25	0.5	2	4
10,006	2	4	2	4	0.13	0.5	4	8
10,007	4	8	2	4	0.25	0.5	4	8
10,008	2	4	4	8	0.25	0.5	4	8
10,009	0.5	1	4	8	0.06	0.13	4	8
10,010	4	8	1	2	0.13	0.25	8	16
10,020	2	4	4	8	0.25	0.5	4	8
10,021	2	4	1	2	0.25	0.5	4	8
10,022	2	4	2	4	0.25	0.5	2	4
10,023	4	4	1	2	0.13	0.25	2	4
10,024	2	4	1	4	0.25	1	4	8
10,025	2	4	1	2	0.06	0.25	4	8
10,026	4	4	4	8	0.5	1	4	8
10,027	4	8	1	2	0.13	0.25	4	8
10,028	2	8	4	16	0.25	0.5	8	16
10,029	2	4	4	8	0.25	0.5	4	8
10,030	4	8	2	4	0.25	1	2	8
10,031	4	8	2	4	0.25	0.5	4	8

MIC, minimum inhibitory concentrations; MFC, minimum fungicidal concentrations; ITZ, Itraconazole; TRB, terbinafine; AMB, amphotericin B. values expressed as μg/mL.

**Table 3:** Antifungal susceptibility profiles of *S. globosa* isolates against In-58 and antifungals.

*Sporothrix* species	Parameter	Drug (μg/mL)			
		In-58	ITZ	TRB	AMB
*S. globosa* (*n* = 21)	MIC Range	0.5–4	1–4	0.13–0.5	2–8
	GM	2.69	1.87	0.20	3.87
	MIC50	4	2	0.25	4
	MIC90	4	4	0.25	8
	MFC Range	1–8	2–16	0.25–1	4–16
	GM	5.21	4.13	0.47	8
	MFC50	4	4	0.5	8
	MFC90	8	8	1	16

### 3.2 Drug interaction assays

Drug interactions assays were performed with 10 *S. globosa* isolates using the checkerboard method. On the basis of the results obtained for the mycelial phase (see Section 3.1), combinations between In-58 and other drugs (ITZ, TRB, and AMB) were applied as shown in Table 4.

**Table 4:** FICI for the combination of In-58 with ITZ, TRB, and AMB against filamentous form of *S. globosa.*

Drug	Parameter	*S. globosa* ID of SHJU									
		10,001	10,003	10,004	10,007	10,020	10,021	10,022	10,026	10,027	10,029
In-58	*MIC combined (ITZ/TRB/AMB)	2/4/2	2/4/1	2/4/1	2/4/1	1/2/0.5	1/2/0.5	1/2/0.5	1/4/2	1/4/1	2/4/2
ITZ	FICI	0.75	0.75	1	1	0.75	0.75	1	0.5	0.5	1
	**IN	IND	IND	IND	IND	IND	IND	IND	**S**	**S**	IND
TRB	FICI	1.5	2	2	1.5	2	2	2	2	2	2
	IN	IND	IND	IND	IND	IND	IND	IND	IND	IND	IND
AMB	FICI	0.75	0.5	0.75	0.75	0.75	0.75	0.5	1	0.75	0.75
	IN	IND	**S**	IND	IND	IND	IND	**S**	IND	IND	IND

FICI, fractional inhibitory concentration index; *MIC expressed as μg/mL; **IN: ≤0.5 synergism (S); 0.5–4.0 indifferent (IND); >4 antagonism (AN).

For In-58 with ITZ and AMB, the FICI values were considered indifferent for eight of the 10 *S. globosa* isolates, and synergetic for two isolates. The FICI values were indifferent for all 10 isolates for the combination of In-58 with TRB. No antagonism was observed in the antifungal activities between In-58 and any of the tested antifungal agents.

### 3.3 Time-kill assays

**Figure 1:**
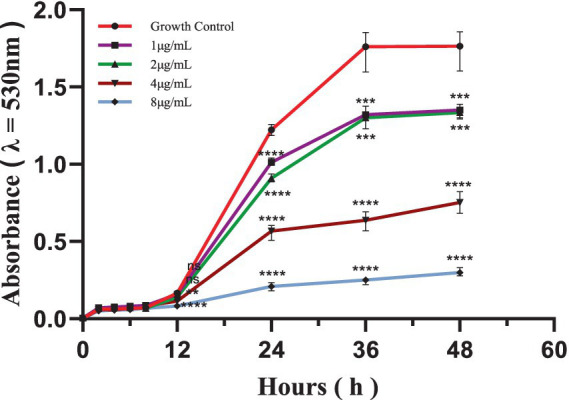
Time-kill curve of In-58 at 1 μg/mL, 2 μg/mL, 4 μg/mL, and 8 μg/mL against *S. globosa* SHJU10001. ** *p* < 0.01, *** *p* < 0.001, **** *p* < 0.0001 compared with GC.

Time-dependent kill curves for *S. globosa* SHJU10001 treated with different concentrations of In-58 (1, 2, 4, and 8 μg/mL) were plotted. In accordance with a previous publication (25), the fungistatic effect was defined as growth inhibition of 50% after 24 h, and the fungicidal effect was defined as killing of 99.99% of fungal cells after 48 h. For the control (no drug), the period from 12 to 24 h was characterized by the exponential growth phase, while between 36 and 48 h the culture entered a plateau phase. Starting from 12 h, compared with the control, the cultures treated with 4 (*p* = 0.001) and 8 μg/mL In-58 (*p* < 0.0001) both showed significant decreases in optical density (λ = 530 nm). In-58 displayed 50% inhibition of *S. globosa* at 4 μg/mL, and 90% inhibition at 8 μg/mL (Figure 1). These concentrations coincided with the antifungal susceptibility results (MIC of 4 μg/mL and MFC of 8 μg/mL), demonstrating the dose-dependent fungicidal activity of In-58 against *S. globosa*.

### 3.4 Micromorphological observation of *Sporothrix globosa* following treatment with In-58

**Figure 2:**
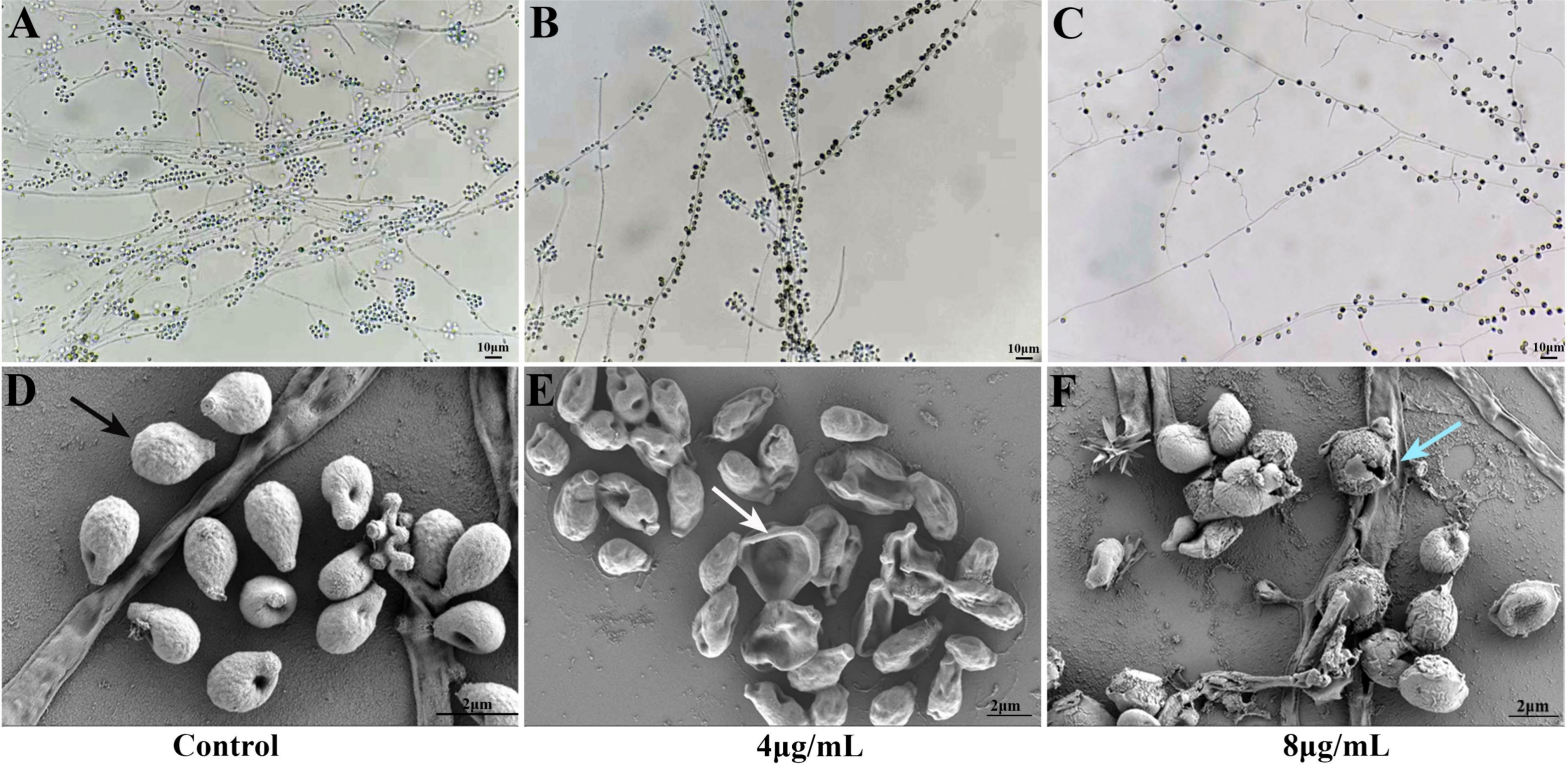
Slide culture of growth control **(A)** and effect of In-58 at 4 μg/mL **(B)** and 8 μg/mL **(C)** on *Sporothrix globosa* SHJU10001. As the concentration of In-58 increased, fungal growth was obviously inhibited. Scanning electron microscopy images of *S. globosa* SHJU10001 exposed to In-58. **(D)** Control group showing cells with a smooth surface (black arrow). **(E)** *S. globosa* cells treated with 4 μg/mL of In-58, showing dented but unruptured cell walls (white arrow). **(F)** *S. globosa* cells treated with 8 μg/mL of In-58, showing alterations in cell wall integrity and cell disruption (blue arrow).

As shown in Figures 2A–C, *S. globosa* not treated with In-58 displayed interwoven septate hyaline hyphae, and clustered small groups of conidia, which distributed alongside hyphae sympodially with floral arrangements. *S. globosa* treated with 4 μg/mL In-58 showed fewer septate mycelial filaments and sleeve-like arrangements of conidia. *S. globosa* treated with 8 μg/mL In-58 exhibited relatively sparse hyphae and conidia. Thus, as the concentration of In-58 increased, conidia of *S. globosa* became less likely to cluster and they were loosely attached to the hyphae, and the typical sleeve-like conidiophores were less obvious, which indicates that In-58 inhibited the growth of *S. globosa*.

Microstructural changes in 4 and 8 μg/mL In-58-treated *S. globosa* cells were observed by SEM. As shown in Figures 2D–F, *S. globosa* cells in the control group were intact with smooth surfaces. Cells in the group treated with 4 μg/mL In-58 were dented, but with no obvious rupture of the cell wall. In the 8 μg/mL In-58 treatment group, irregular shapes, squeezed, depressed cell surfaces, and the release of intracellular components were observed, indicating the disruption of *S. globosa* cells.

### 3.5 PI staining

**Figure 3:**
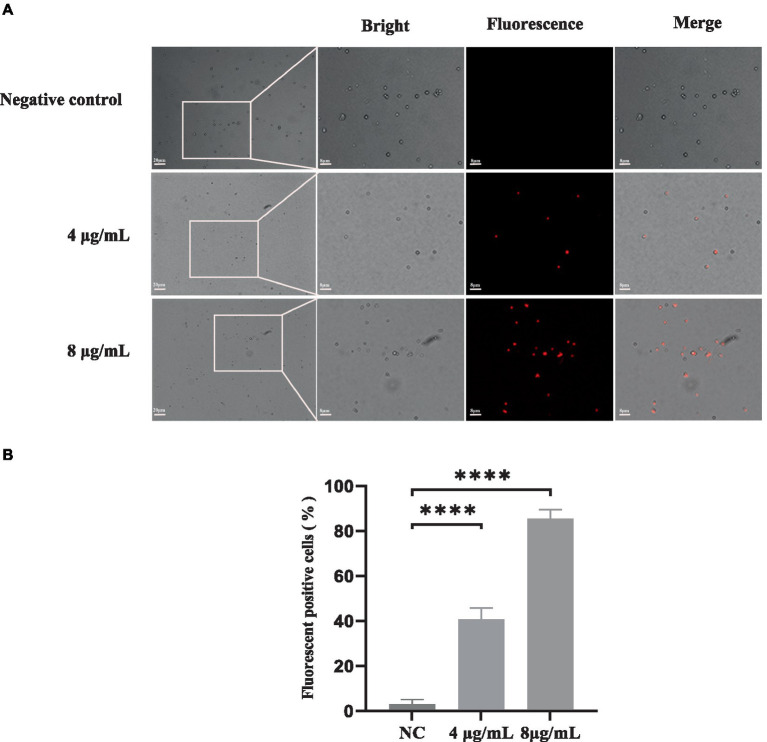
The uptake of propidium iodide by *S. globosa* SHJU10001. **(A)** Fungal cells were treated with 4 or 8 μg/mL In-58. The negative control was treated with PBS, used to examine *S. globosa* membrane integrity. The displayed figures are the most representative results selected from the experiments. **(B)** Percentage of fluorescence-positive cells. NC, negative control. **** *p* < 0.0001.

PI is a fluorescent dye that can only penetrate into cells with compromised membranes; it then binds to DNA by intercalating between bases, resulting in bright red fluorescence. PI staining was performed to analyze the plasma membrane integrity of *S. globosa* cells following treatment with In-58 (4 or 8 μg/mL) for 12 h. Three fields were randomly selected to quantify the number of fluorescence-positive cells for each condition. NC group: PI positive cells were 1, 2, 2 out of 75, 80, 73, respectively. 4 μg/mL In-58 group: PI positive cells were 25, 30, 28 out of 60, 72, 70, respectively. 8 μg/mL In-58 group: PI positive cells were 86, 90, 76 out of 100, 105, 91, respectively. The number of fluorescence-positive cells increased with elevated In-58 concentration (Figure 3). The percentage of fluorescence-positive cells significantly increased (*p* < 0.0001) for In-58 at both 4 and 8 μg/mL when compared with the negative control. These findings demonstrate that In-58 damages the plasma membrane of *S. globosa* cells in a dose-dependent manner.

Collectively, the results of fungal inhibition, SEM and PI uptake assays indicate that In-58 disrupts the integrity of the *S. globosa* cell wall, resulting in cell death.

### 3.6 Intracellular ROS accumulation

**Figure 4:**
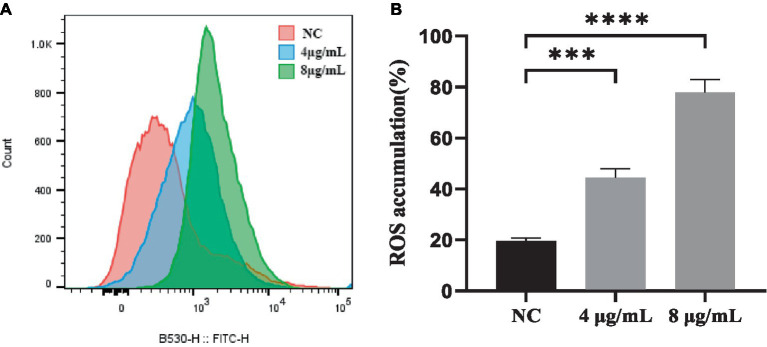
**(A)** Flow cytometry was used to detect reactive oxygen species accumulation in *S. globosa* SHJU10001 treated with 4 or 8 μg/mL In-58. The negative control was treated with PBS. **(B)** A highly significant difference was observed between the control group and each In-58-treatment group. *** *p* < 0.001, **** *p* < 0.0001.

ROS generation is a downstream consequence of the stress response to drug-mediated disruption of target processes. To determine if the killing mechanism of *S. globosa* cells by In-58 was ROS-related, the ROS levels in *S. globosa* cells were analyzed by flow cytometry in which 50,000 events for each case were analyzed following incubation with In-58 (4 or 8 μg/mL) for 12 h. As shown in Figure 4, ROS accumulation in the In-58-treated groups was significantly elevated compared with the negative control.

### 3.7 MMP

**Figure 5:**
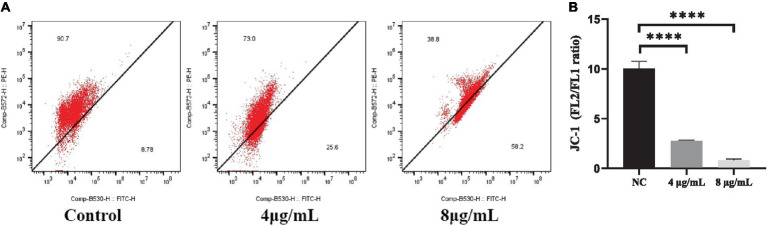
**(A)** Flow cytometry was used to assess the mitochondrial membrane potential in *S. globosa* SHJU10001 treated with 4 or 8 μg/mL In-58. The negative control was treated with PBS. **(B)** A highly significant difference was observed between the control group and the each In-58-treatment group. The displayed figures represent are the most representative results selected from the experiments. **** *p* < 0.0001.

The JC-1 staining assay was performed to determine the effect of In-58 on the MMP of *S. globosa*. In healthy cells, JC-1 dye enters mitochondria with normal membrane potential and forms red-fluorescent aggregates because of the high membrane potential inside, while in apoptotic cells where the membrane potential is decreased, JC-1 remains in its green-fluorescent monomeric form because it fails to accumulate at a concentration necessary for aggregate formation. This difference in fluorescence between healthy and apoptotic cells provides a method to assess mitochondrial function and cell viability. The ratio of the fluorescence intensities of red aggregates of JC-1 (FL2) to green monomers (FL1) was calculated for In-58-treated *S. globosa* cells. The percentage of cells with red aggregates of JC-1 was 90.7% in the control group, 73% in the 4 μg/mL In-58-treatment group, and 38.8% in the 8 μg/mL In-58-treatment group (Figure 5A); that of cells with green monomers of JC-1 was 8.78% in the control group, 25.6% in the 4 μg/mL In-58-treatment group, and 58.2% in the 8 μg/mL In-58-treatment group. Thus, the FL2/FL1 ratio decreased significantly in both the 4- and 8 μg/mL-treatment groups compared with the control (Figure 5B), suggesting a dose-dependent decrease of MMP after In-58 treatment.

In-58-induced oxidative stress thus caused depolarization of the mitochondrial membrane of *S. globosa*, which would lead to mitochondrial dysfunction.

### 3.8 *Galleria mellonella* survival assays

**Figure 6:**
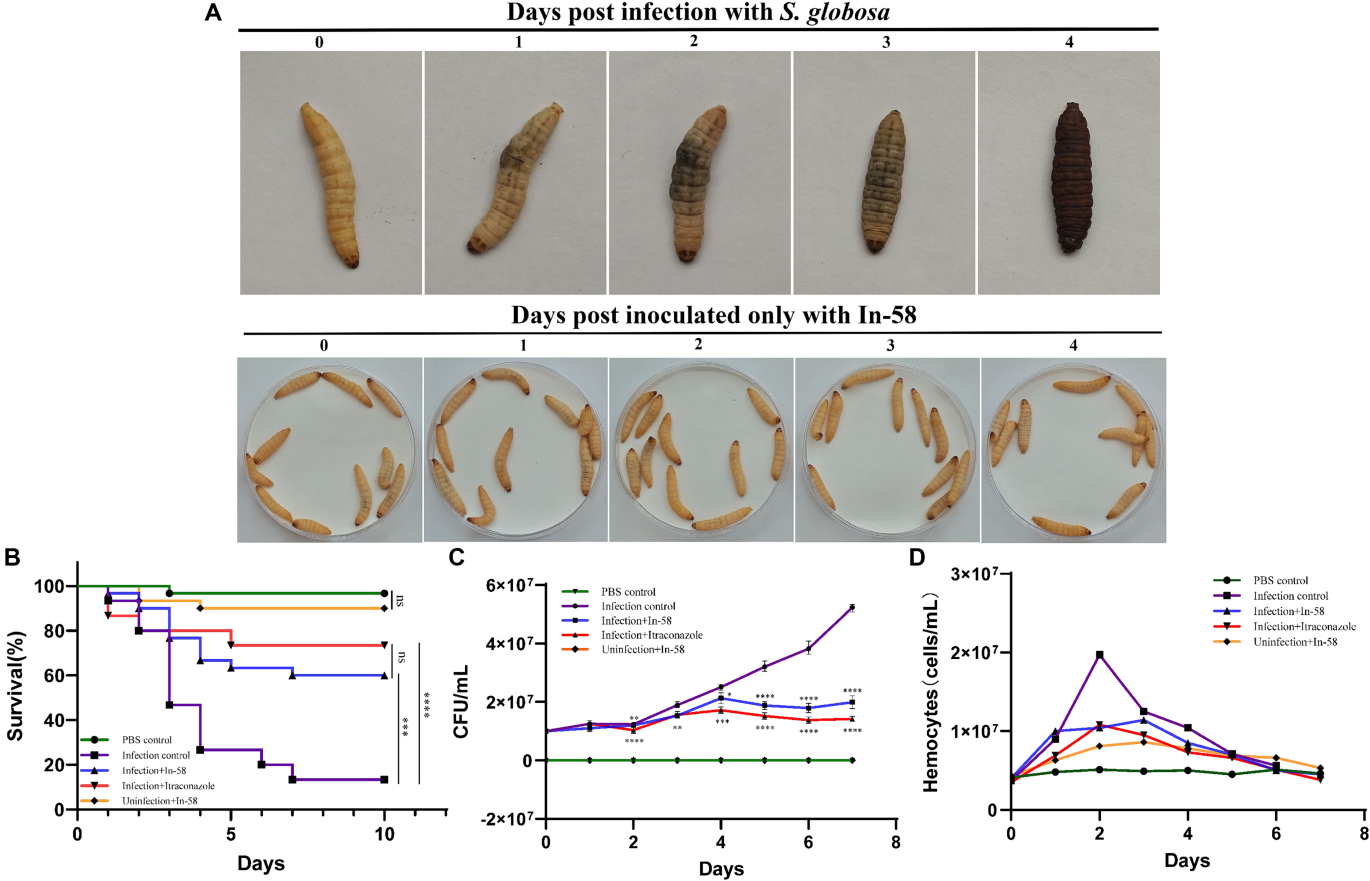
*Galleria mellonella* larvae as a model for infection with *S. globosa* SHJU10001. **(A)** The melanization of *G. mellonella* during infection with 1 × 10^6^ conidia/larva of *S. globosa*. Larvae became progressively darker over the course of the infection. Larva inoculated only with In-58 mostly survived and remained in good health. **(B)** Survival curves during 10 days infection. **(C)** Fungal burden determined in the process of infection. **(D)** Hemocyte density of *G. mellonella* in different groups. * *p* < 0.05, ** *p* < 0.01, *** *p* < 0.001, **** *p* < 0.0001.

The *in vivo* effect of the exposure to In-58 was evaluated using the invertebrate *G. mellonella.* As shown in Figure 6A, *G. mellonella* larvae became progressively darker (i.e., melanized) over the course of infection with *S. globosa* while larvae inoculated only with the In-58 peptide showed no signs of melanization, and most of them remained healthy and alive.

The toxicity of In-58 was evaluated in larvae after exposure to a single dose (5 mg/kg of body weight) without infection (Group V – see Section 2.10). Little mortality was observed (90% of the larvae survived). This was similar to the PBS-inoculated control group (Group I), in which 96.7% of the larvae survived (no significant difference).

In the infection control group (Group II, infection of larvae with *S. globosa*, no drug treatment), a larval survival rate of 13.3% was observed after 10 days. In the infection + In-58 treatment group (Group III), the survival rate was 60% (Figure 6B). In the infection +20 mg/kg ITZ treatment group (Group IV), the survival rate was 73.5% (not significantly different from that in the infection + In-58 treatment group). The enhanced survival rates in the In-58 and ITZ treatment groups compared with the infection control group were highly significant by the log-rank test (Figure 6B).

### 3.9 Determination of fungal burden from infected *Galleria mellonella*

In our study, hemolymph was collected daily, and the diluted suspension was cultured on PDA-plates. The resulting colonies were then examined by LPCB staining under a microscope. Colonies exhibiting characteristics such as septate hyphae and conidia organized in structures resembling flowers were identified as belonging to the genus *Sporothrix*.

Figure 6C shows the evolution of the fungal burden along the survival curve. As expected, no fungal burden was detected in the PBS or In-58 control groups (Groups I & V). The fungal burden increased from 1 × 10^7^ to 5.21 × 10^7^ CFU/mL in the infection control group (Group II) from day 0 to day 7. In the In-58-treated infected larval group (Group III), the fungal burden increased from 1 × 10^7^ CFU/mL on day 0 to 1.99 × 10^7^ CFU/mL on day 7. In the ITZ-treated infected larval group (Group IV), the fungal burden increased from 1 × 10^7^ CFU/mL on day 0 to 1.42 × 10^7^ CFU/mL on day 7. A highly significant difference was revealed between the PBS control group and the In-58/ITZ treatment groups from day 2 to the end of the experiment. A significant difference was also observed between Groups III and IV, indicating that ITZ showed better antifungal activity than In-58 in this experiment.

### 3.10 Enumeration of hemocytes from *Galleria mellonella*

Fluctuation of hemocyte density was observed by enumerating these cells for 7 days post-infection. In the PBS control group (Group I), the hemocyte count fluctuated around 5 × 10^6^ cells/mL during the 7 days experiment. In the In-58 control group (Group V), the hemocyte density increased from 4.3 × 10^6^ cells/mL at 0 h to 8.6 × 10^6^ cells/mL at 72 h, then decreased to 5.3 × 10^6^ cells/mL at day 7. In the infection control group (Group II), the hemocyte count increased from 3.7 × 10^6^ cells/mL at 0 h to 1.97 × 10^7^ cells/mL at 48 h, which was the highest value observed in any group, then decreased to 5.6 × 10^6^ cells/mL at day 7. In the group treated with In-58 post-infection (Group III), the hemocyte count increased from 4 × 10^6^ cells/mL at 0 h to 1.14 × 10^7^ cells/mL at 72 h, then decreased to 4.5 × 10^6^ cells/mL at day 7; in the ITZ-treated group (Group IV), it increased from 3.5 × 10^6^ cells/mL at 0 h to 1.08 × 10^7^ cells/mL at 48 h, and decreased to 3.8 × 10^6^ cells/mL at day 7. Thus, overall, in the infection control and drug-treated post-infection groups, the hemocyte density was found to increase prominently at 24 h post-infection, reach its peak at 48–72 h, then decline significantly (Figure 6D). These data demonstrate the ability of *G. mellonella* hemocytes to respond quantitatively to *S. globosa* infection and treatment afterwards.

### 3.11 Histopathological characteristics of *Galleria mellonella*

**Figure 7:**
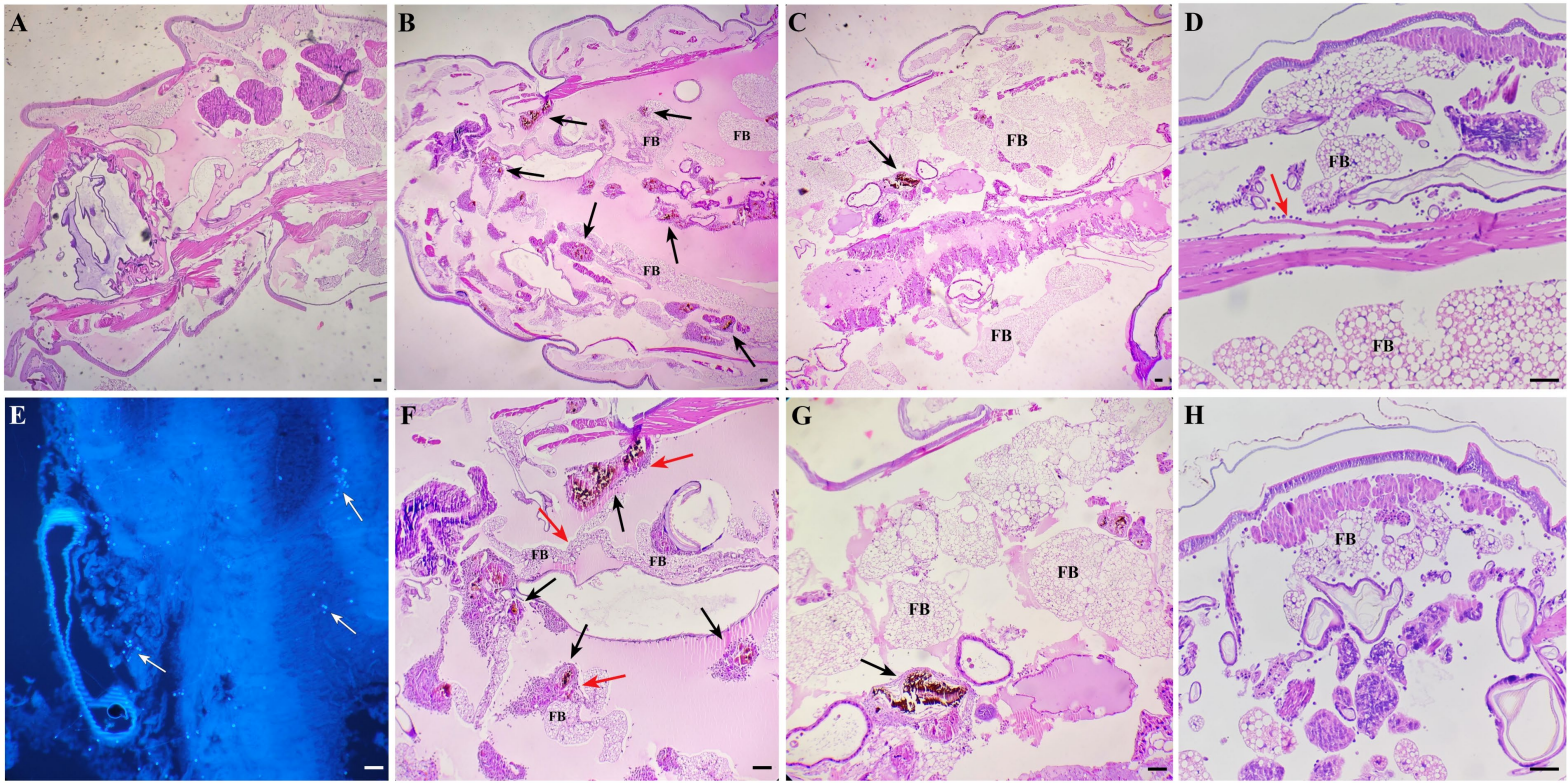
Histopathological results for *S. globosa*-infected *G. mellonella* larvae using hematoxylin and eosin and Calcofluor-White staining. **(A)** Control group (phosphate-buffed saline, 4× magnification). **(B,F)** Infection group (10^6^ CFU of *S. globosa*), which displayed aggregation of melanization (black arrow) and hemocytes (red arrow) within the tissue 24 h post-infection (4×, 10× magnification). Infected larvae treated with In-58 **(C,G)** looked healthy, with a few individually distributed hemocytes exhibiting no obvious aggregation and little melanization at 24 h (4×, 10× magnification). **(E)** Fungal elements (white arrow) were observed in the infection control group. **(D)** In the infection control group, aggregates of hemocytes (red arrow) were observed surrounding the muscle fibers and **(F)** the inoculum site at 24 h; then **(H)**, hemocytes were diffusely distributed at 48 h. FB, fat body; scale bar = 20 μm.

Larvae of *G. mellonella* each infected with 10^6^ CFU of *S. globosa* were placed at 35°C, then tissue was collected and histopathological analysis was performed at 24 and 48 h post-infection. Images for the PBS control group (Group I) are shown in Figure 7A, in which clean tissue architecture was observed and hemocytes were distributed mainly in the subcuticular area and dispersed in the fat body. Compared with the PBS control group, in the infection control group (Group II) 24 h after inoculation, hemocytes were recruited at the infection site and spindle cells aggregated in melanization nodules, entrapping fungal elements (Figures 7B,F). Round hemocytes changed to a spindle shape to stratify around the pathogen in the external part of melanization nodules, thus emphasizing their role in encapsulation (35). In contrast, infected larvae treated with In-58 (Group III) looked healthy, with little melanization and a few individually distributed hemocytes exhibiting no obvious aggregates at 24 h (Figures 7C,G). Fungal elements were observed in the infection control group stained with CFW (Figure 7E). Interestingly, as infection progressed, aggregates of hemocytes were observed surrounding the muscle fibers at 24 h in the infection control group (Figure 7D), then hemocytes were diffusely distributed at 48 h (Figure 7H).

## 4 Discussion

**Figure 8:**
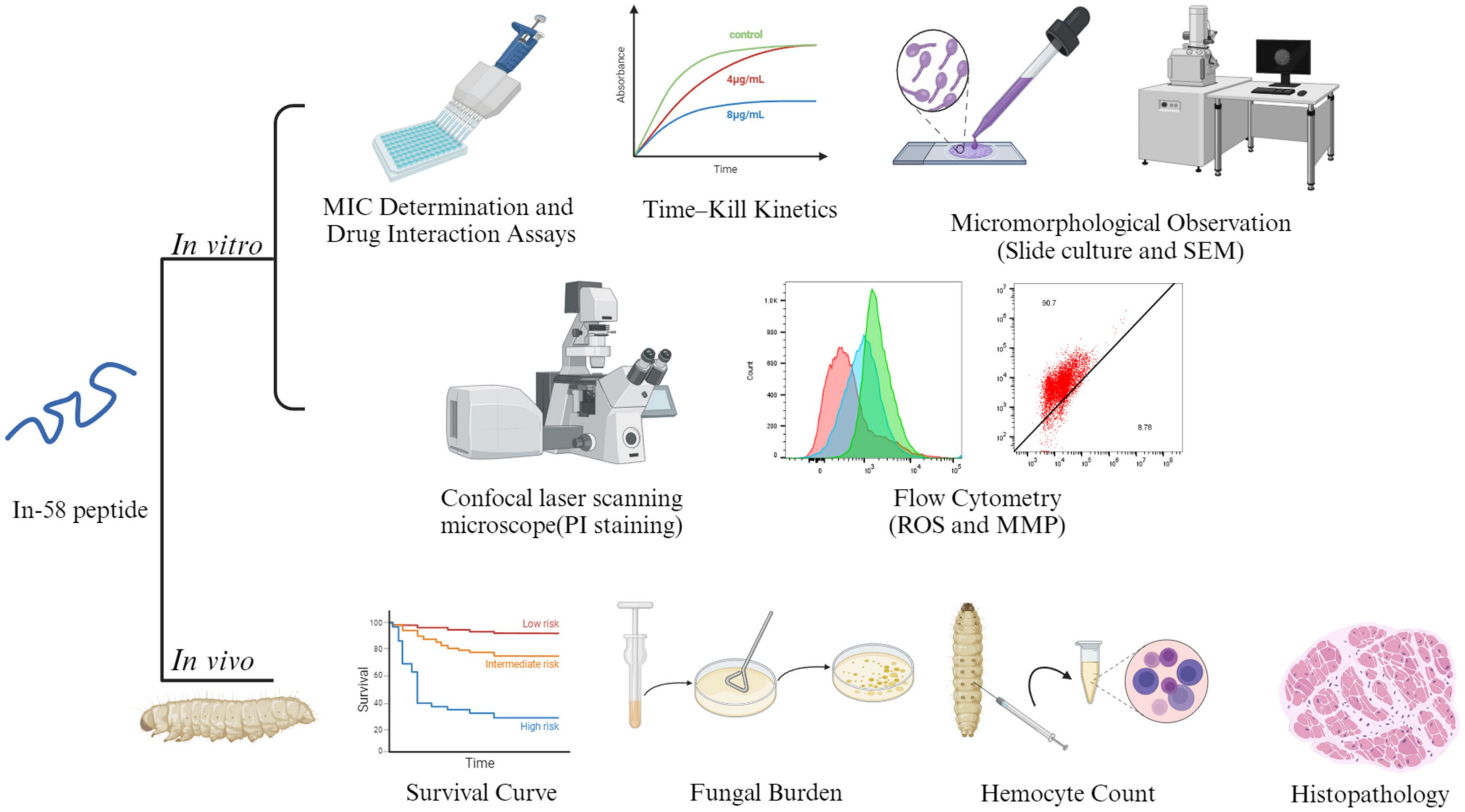
A graphic model of the *in vitro* and *in vivo* experiments of In-58. Images of this figure were created using http://BioRender.com.

Indolicidin, which was initially extracted from cytoplasmic granules of bovine neutrophils, has been proven to be an outstanding wide-spectrum AMP, but high hemolytic activity limits its application (36). Indolicidin has a wide range of biological targets, including Gram-positive bacteria, Gram-negative bacteria, fungi and parasites. It is active not only against planktonic cells of organisms, but also against biofilms, which are more difficult to eliminate (37, 38). Compared with conventional antibiotics, indolicidin exhibits increased resilience against microbial drug resistance; for example, it is effective against multidrug resistant-enteroaggregative *Escherichia coli* (MDR-EAEC) (39). In terms of its antifungal activity, it has been demonstrated to be active against *C. albicans*, with a MIC of 12.5–50 μg/mL (40). Many indolicidin analogs have been synthesized, purified, and evaluated. In-58 was created by substituting all the tryptophan residues in In-58 with D-phenylalanine. In-58 shows both good antimicrobial activity and low hemolytic activity (20). Previously, In-58 was tested against several bacteria, including *E. coli*, *Staphylococcus* (*Staph*.) *aureus*, *Pseudomonas aeruginosa*, and *Bacillus subtilis*, and displayed an average MIC50 < 10 μg/mL and minimal concentration inducing 50% hemolysis >200 μg/mL (20). Resistance to antifungal agents has become a major problem in the treatment of fungal infectious diseases. An increasing immunocompromised population limited new antifungals, wide-scale global travel, fungal dispersion in the environment, and fungal biofilm formation result in the emergence of drug-resistant fungi (41). *C. auris* shows resistance to AMB, voriconazole, fluconazole, and caspofungin, while *Candida* and *Aspergillus* spp. decreasingly respond to azole drugs (42). Some *Sporothrix* isolates have developed resistance to ITZ, the standard treatment for sporotrichosis (43). Given the limitations of current treatments, novel agents are under development to address resistance to the available antifungal drugs for sporotrichosis (44). Taking all of these factors into account, In-58 exhibits great potential for use against microbial infection. Here, we investigated its antifungal activity against *S. globosa in vitro* and *in vivo* (Figure 8).

The MIC of In-58 against *S. globosa* isolates (*n* = 21) ranged from 0.5 to 4 μg/mL in broth microdilution assays (MIC50 4 μg/mL), while the MFC ranged from 1 to 8 μg/mL (MFC50 4 μg/mL). These values were higher than those described for *Listeria monocytogenes* (MIC 0.75 μg/mL), but a little lower than those reported for *Bacillus cereus* (MIC 6.25 μg/mL) and *Staph. aureus* (MIC 6.25 μg/mL) (20). Other AMPs, such as ToAP2D, were also reported to exert antifungal activity against *S. globosa* (MIC 156.25 μg/mL) (28). Compared with bacteria, fungi are often more difficult to eliminate, possibly because the cell wall of fungi consists of chitin, which plays a crucial role in stability limiting the access of molecules to the plasma membrane (45). Thus, this protective barrier of fungi also makes it difficult for In-58 to exert antifungal activity.

Checkboard assays showed no antagonistic effect between In-58 and common antifungal drugs (ITZ, TRB, or AMB). In-58 acted synergistically with ITC and AMB against 20% of our *S. globosa* isolates and the interaction was indifferent for 80% of the isolates; it was also indifferent for all isolates with TRB. A few antifungal drugs have been reported to be synergistic with AFPs. *C. albicans* was tested most in combination with AFPs and antifungal agents, and synergies occurred with AMB (46). Lactoferricin B, IB367, and hepcidin-20 were, respectively, reported to wok synergistically with ITZ, TRB, and AMB against different fungi (47–49). The activity of antifungals usually involves inhibition of the synthesis of the fungal cell wall, and AFPs might achieve this by disrupting the structure directly. Therefore, when AFPs and other drugs act together on fungi, they may exhibit a synergistic effect. The carpet-like, barrel-stave, and toroidal models that are used to describe the pores AMPs generate in bacterial membranes are also applicable for AFPs acting on fungal membranes (17). It can be hypothesized that In-58 damages the cell wall of fungi, causing the death of fungal cells. This was supported by our SEM and PI-uptake assays, in which the effects of In-58 were dose-dependent. When the peptide inserts into fungal cells, the permeability of the membrane is altered, resulting in ROS accumulation and the activation of stress-response pathways (50). Additionally, some AFPs have been demonstrated to interact with the content of the cell membrane or with enzymes engaged in production of membrane components. For instance, RsAFP2, a plant defensin isolated from radish seed, was found to interact with fungal glucosylceramides. Aureobasidin A isolated from the fungus *Aureobasidium pullulans* has an impact on inositol phosphorylceramide synthase, which is necessary for sphingolipid biosynthesis (51, 52). Mechanisms of action of indolicidin have been proposed, including transient membrane perturbation, interaction with intracellular targets such as inhibition of proteins and DNA synthesis, and an anion carrier mechanism (53). These might be similar to the mechanisms of action of the indolicidin derivative In-58.

Apoptosis includes intrinsic and extrinsic pathways, with the intrinsic pathway being primarily associated with mitochondria. One hallmark of apoptosis is impaired mitochondrial function, and decreased MMP is an early event in apoptosis (54). A high level of ROS depolarizes the mitochondrial membrane, leading to a lower MMP. The ROS accumulation and depolarization of the mitochondrial membrane are vital in the apoptosis of fungi (55). ROS mainly originate from mitochondria, where they are produced primarily through the respiratory chain, also marking an early event in apoptosis. Here, *S. globosa* displayed significantly elevated ROS accumulation after incubation with In-58. We hypothesized that the antifungal effects of In-58 may in part be attributable to ROS-mediated oxidative stress. To confirm this mechanism, the cationic dye JC-1 was used to examine alterations in the MMP. We found a decreased ratio for FL2/FL1 for In-58-treated *S. globosa* (Figure 5), suggesting the decrease of MMP, which demonstrates that In-58 may hold antifungal activity against *S. globosa* by generating excessive ROS and depolarizing the mitochondrial membrane. A study on melittin triggering apoptosis in *C. albicans* also showed remarkable accumulation of ROS within cells and decreased MMP (56). It is notable that mitochondria release ROS during apoptosis, and excessively high levels of ROS further promote apoptosis, forming a positive feedback loop (57).

To investigate *S. globosa* infection and the anti-infective ability of In-58 *in vivo*, *G. mellonella* was used as a model; this organism shares a similar innate immune response to pathogens with mammals (58). Recent study has shown that experimental results using *G. mellonella* as the infection model are highly correlated with those for mammals such as mice (59). *G. mellonella* has been used in a growing number of publications for evaluation of novel antimicrobial drugs, including AMPs (60, 61). Injection of NapFFKK-OH peptide after infection of the *G. mellonella* model for 24 h with *Staph. epidermidis*, *Staph. aureus*, *E. coli* or *P. aeruginosa* led to at least a 2 log_10_ CFU/mL reduction in bacterial counts for all the pathogens (62). The synthetic AMP IKR18 demonstrated anti-infective activity against *Staph. aureus*, methicillin-resistant *Staph. aureus*, and *Acinetobacter baumannii* in *G. mellonella* (63). The larvae can be incubated at 35°C, are easy to manipulate, and dosing is relatively precise, making this model system an excellent choice for our study. In our pilot experiment, 10^6^ CFU of *S. globosa* per larva was found to be the optimum lethal dose.

After applying In-58 to infected larvae, a significant increase in survival was observed compared with the infection control group. There was no significant difference in larval survival between the infected then In-58-treated group and the group treated with the standard antifungal drug ITZ. The effectiveness of some other AMPs was also evaluated by observing survival of *G. mellonella*. For instance, indolicidin was reported to exert excellent antimicrobial activity against MDR-EAEC, with a significantly increased survival rate of treated larvae, which the authors deduced might be due to a better bioavailability of indolicidin in larvae than antibiotics meropenem (39). In our experiments, the survival rate in the In-58-treated but not infected control group (Group V) serves an indicator of *in vivo* safety of the peptide. Thus, In-58 possesses efficacy against *S. globosa* as well as favorable safety *in vivo*. The improved cytotoxicity of In-58 compared with that of indolicidin may be due to changes in the net charge and hydrophobicity of the peptide (20).

The fungal burden of hemolymph was examined to monitor dynamic changes during infection of *G. mellonella* by *S. globosa*. In-58 inhibited fungal growth, but ITZ exhibited better antifungal activity than In-58 at the doses applied. Meanwhile, hemocyte density was found to increase prominently at 24 h post-infection, peak by 48–72 h, then decline significantly, reflecting the ability of insect hemocytes to respond quantitatively to *S. globosa* infection and treatment. In *G. mellonella*, plasmatocytes and granular cells are involved in most cellular defense responses (64). An increase of hemocytes represents the cellular immune response of the larvae to the elevated burden of *S. globosa*, and one study has suggested that the function of insect hemocytes might be similar to those of human macrophages and neutrophils (65). The decline of hemocytes from day 3 post-infection in our experiments might be a consequence of fungal cytotoxic activity toward the host (66). Description of changes in hemocyte counts of *G. mellonella* larva infected with different pathogens varies in different studies as the infection progresses. The hemocyte density of larva infected with MDR-EAEC was found to increase significantly at 6 h post-infection, reach its peak by 12 h, and decline significantly thereafter (39). In another study, circulating hemocytes increased at day 1 post-infection with *S. brasiliensis*, dropped at day 3, then increased gradually in the following days; at day 7, a prominent rise was recorded with no decline until the end of observation (32).

Here, histopathology of *G. mellonella* was used to study the changes associated with host–pathogen interaction and migration of hemocytes during the infection with *S. globosa* and treatment. Aggregation of hemocytes around muscle fiber changed to diffuse distribution from 24 to 48 h post-infection, indicating that hemocytes may be recruited toward the inner part of the larva, such as the heart region and gut wall, aiming at eliminating fungal cells, as reported previously (35, 39). Melanization was obviously disseminated in the infection control group, while infected larvae treated with In-58 looked healthy with only a few distributed hemocytes exhibiting no obvious aggregates and little melanization at 48 h. In other fungal infection models of *G. mellonella*, melanized nodules were also observed, as well as hemocytes that changed into a spindle form to trap the pathogen at the infection site (35). Melanized nodules can be observed in different kinds of fungal infection within larvae, for example in *G. mellonella* infected with *Mucor circinelloides* and *C. auris* (35, 67, 68). As hemocytes in hemolymph of *G. mellonella* is similar to neutrophils in mammalian blood, and fluctuation of hemocyte density is part of the innate immune response of the larvae to fungal infection, and decreased melanization may be relevant to the clearance of fungi from the host, which is likely to link to human immune response against *S. globosa* infection by phagocytose and kill pathogens via production of superoxides (69).

In conclusion, In-58 is active against *S. globosa in vitro* and *in vivo*. Further investigations are needed to understand the precise mechanisms of its function, so that it might serve as a novel therapeutic for the treatment of sporotrichosis, alone or in combination with other antifungal drugs.

## Data Availability

The original contributions presented in the study are included in the article, further inquiries can be directed to the corresponding authors.
